# Is There Still a Place for Forceps Delivery in Modern Obstetrics?

**DOI:** 10.34763/jmotherandchild.20232701.d-23-00057

**Published:** 2023-11-03

**Authors:** Katarzyna Zając, Małgorzata Rybnik, Marcin Kęsiak, Jarosław Kalinka

**Affiliations:** Department of Perinatology, Medical University of Lodz, Poland; Department of Neonatology and Intensive Neonatal Care, Pirogow Hospital, Lodz, Poland

**Keywords:** obstetric forceps, operative vaginal delivery, maternal outcomes, neonatal outcomes

## Abstract

**Background:**

Nowadays, we are witnessing a decrease of vaginal instrumental deliveries and continuous increase of caesarean section rate. However, proper identification of possibility of execution, indications for instrumental delivery and their skilful use may improve the broadly understood maternal and neonatal outcomes. The aim of this study is to present prevalence, risk factors, indications and outcomes of forceps deliveries among the patients at Department of Perinatology, Lodz.

**Material and methods:**

A retrospective study was conducted at the Department of Perinatology, Medical University of Lodz. The study included forceps deliveries carried out between January 2019 and December 2022. Total number of 147 cases were analysed in terms of indications for forceps delivery and maternal and neonatal outcomes such as vaginal – or cervical – laceration, postpartum haemorrhage, perineal tear, newborn injuries, Apgar score, umbilical cord blood gas analysis, NICU admission and cranial ultrasound scans.

**Results:**

The prevalence of forceps delivery was 2.2%. The most common indication for forceps delivery was foetal distress (81.6%). Among mothers, the most frequent complication was vaginal laceration (40.1%). Third-and fourth-degree perineal tears were not noted. Regarding neonatal outcomes, Apgar score ≥ 8 after 1st and 5th minute of life received accordingly 91.2% and 98% of newborns. Only 8.8% experienced severe birth injuries (subperiosteal haematoma, clavicle fracture).

**Conclusions:**

Although foetal distress is the most common indication for forceps delivery, the vast majority of newborns were born in good condition and did not require admission to NICU. Taking into consideration high efficacy and low risk of neonatal and maternal complications, forceps should remain in modern obstetrics.

## Introduction

Nowadays, we are witnessing a decrease of vaginal instrumental deliveries [1] and continuous increase of caesarean section – including those performed on fully dilated cervix [2,3]. However proper identification of possibility of execution, indications for instrumental delivery and their skilful use may improve the broadly understood maternal and neonatal outcomes as well as shorten convalescence for mother and child [4]. Two most popular of the above-mentioned techniques are obstetric forceps and vacuum. In this study, we focus on tools that offer a higher success rate and enable faster delivery of the baby, which are forceps [5,6].

The aim of this study is to present prevalence, risk factors and indications for forceps delivery as well as maternal and neonatal outcomes among the patients at Department of Perinatology of Medical University of Lodz (Poland) between January 2019 and December 2022.

## Material and methods

This retrospective study was conducted at the Department of Perinatology of Medical University of Lodz (Poland). The study included 147 births by forceps delivery carried out between January 2019 and December 2022. Among the mentioned, three of the forceps deliveries were used in twin pregnancies in order to deliver the second twin.

Data were obtained on the basis of electronic and paper documentation of mothers and newborns. Maternal demographic recorded age, gestational age, parity and comorbidity. Delivery was reviewed in terms of application of labour induction, use of epidural anesthesia, length of second stage of labour and indications for forceps delivery. Maternal outcomes of interest were blood loss during labour, vaginal and cervical laceration, postpartum haemorrhage, additional perineal tear, vulvovaginal haematoma, pubic symphysis separation and hospitalisation period after delivery. Neonatal outcomes of interest were Apgar score, birth weight, umbilical cord blood gas analysis, neonatal intensive care unit administration, injuries and cranial ultrasound scans.

Data were collected from the hospital register. Statistics were made with the usage of Microsoft Office Excel. Each parameter was calculated and thoroughly analysed.

## Results

### General information

**Graph 1.**
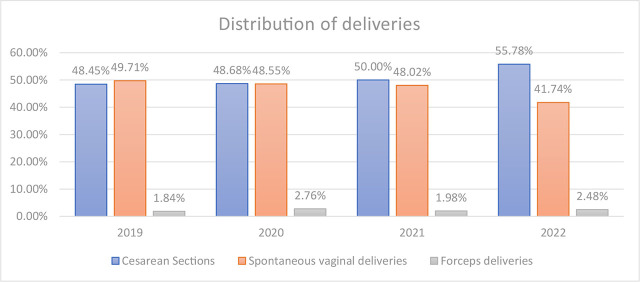
Distribution of deliveries in Department of Perinatology Medical University of Lodz.

During the analysed period of time in our Department of Perinatology, we had a total number of 6604 deliveries – among those 147 (2.2%) were forceps deliveries. In all cases, outlet forceps were used in the second stage of labour. The percentage distribution of deliveries – forceps, spontaneous vaginal and caesarean section – is presented in Graph 1.

### Maternal demographic and outcomes

Within the reviewed patient group age ranged between 17 and 44 – the average age was 30 years: 72.8% (n = 107) were between 26 and 35 years old, and 5.4% (n = 8) patients were over 40 years old.

**Graph 2.**
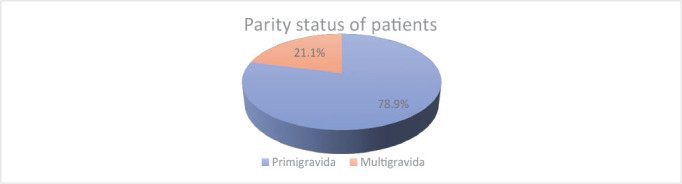
Parity status of patients.

Majority of mothers (116 patients – 78.9%) who required forceps delivery were primigravidas (Graph 2). In the studied group, 5.4 % (8 cases) of forceps delivery were performed during premature deliveries (two of which were cases of twin delivery) with gestational age between 34 and 36 weeks of pregnancy.

In the analysed deliveries, several comorbidities were found among the patients, as shown in Table 1.

**Table 1.** Comorbidities in analysed group.

**Comorbidity**	**Number of patients (n)**	**Percent (%)**
Overweight (BMI = 25,0–29,9)	80	54.4%
Obesity (BMI ≥ 30)	36	24.5%
Diabetes	21	14.3%
Hypertension	21	14.3%

Forty-seven (32%) of the analysed patients underwent delivery induction. Premature rupture of membranes occurred in 27 cases (18.4%). Among patients, 92 (62.6%) had epidural anesthesia during labour, which, in the absence of contraindications, was administered at the patient's request.

The most common indication for forceps delivery in the second stage of labour was foetal distress (n = 120; 81.63 %). Other indications for forceps delivery presented at our hospital were maternal exhaustion (n = 19; 12.92%) and prolonged second stage of delivery (n = 8; 5.44%).

The most frequent maternal complications were vaginal- (n = 59; 40.1%) and cervical- (n = 24; 16.3%) laceration. Other complications occurring with less frequency included postpartum haemorrhage (defined as blood loss of 500 ml or more), additional perineal tear, vulvar haematoma and pubic symphysis separation (Table 2). No third- or fourth-degree perineal tear was reported in the study group; however, in our department, episiotomy is performed in all cases of forceps delivery.

**Table 2.** Maternal complications after forceps delivery.

**Maternal compications**	**Number of patients (n)**	**Percentage (%)**
Vaginal laceration	59	40.1%
Cervical laceration	24	16.3%
Postpartum haemorrhage	21	14.3%
Additional perineal tear	4	2.7%
Vulvovaginal haematoma	2	1.4%
Pubic symphysis separation	2	1.4%

### Foetal outcomes

In terms of the neonatal outcomes, the majority of neonatal birth weight (n = 66; 44.9%) ranged between 3000 and 3500 g at birth. The minimum birthweight was 2100 g, and the maximum was 4340 g. Three of the newborns were large for gestational age, while 16 of them were small for gestational age.

The general condition of each newborn was assessed according to the Apgar score after the 1^st^ and the 5^th^ minute of life. The vast majority of them (n = 134; 91.2%) received the 1^st^ min Apgar score of more than 7 points, what means good general condition after the birth. The status of those who received equal or less than 7 Apgar's points improved and after the 5^th^ minute of life only three (n = 3; 2%) of them were scored below 7.

In the majority of forceps deliveries (n = 117; 79.6%), a blood gas analysis from the umbilical artery was performed. There were five patients (4.3%), whose pH result was lower than 7.0 (Table 3). A base excess (BE) less than −16 was present in eight patients (6.8%). Although a foetal distress was the most common indication, 41 (35%) of newborns had no signs of hypoxia in umbilical artery blood gas analysis (both: pH ≥ 7.2 and BE > −12). Seventy-one (60.7%) babies had the umbilical artery pH between 7.0 and 7.2. Only one newborn met other parameters (such as the Apgar score and neurological symptoms according to the Thompson's scale) and was transferred to the reference center of the therapeutic hypothermy (Polish Mother Memorial Hospital in Lodz), but was ultimately not qualified for the cooling treatment. Other 11 newborns (7.5 %) were admitted to the Neonatal Intensive Care Unit (NICU) because of their general condition just after birth or within the first two hours. Five of them required noninvasive ventilation.

**Table 3.** Results of umbilical artery pH and BE of newborns after forceps delivery.

**Umbilical artery pH**	**Number of patients (n)**	**Percentage (%)**
< 7.0	5	4.3
7.0–7.099	20	17.1
7.1–7.199	51	43.6
≥ 7.2	41	35.0

**Umbilical artery BE**	**Number of patients (n)**	**Percentage (%)**
≤ (−16)	8	6.8
(−15.9) – (−12)	18	15.4
> (−12)	91	77.8

In total, 36 (24.4 %) of babies had any kind of birth trauma after forceps. The most common birth injury (n = 23; 15.6%) among neonates was skin damage (abrasions, lacerations) on the newborn's head. None of them required surgical intervention and the symptoms usually disappeared within the first few days of life. Thirteen newborns (8.8%) experienced more severe birth injuries: subperiosteal haematoma (n = 8; 5.4%), clavicle fracture (n = 3; 2.0%) and conjunctival haematoma (n = 2; 1.4%). One baby was transferred to the Polish Mother Memorial Hospital in Lodz due to an extensive subperiosteal haematoma and symptoms of facial nerve palsy. Medical imaging techniques (CT scans and MRI) showed intracranial and subgaleal haemorrhage and the occipital bone's fracture. There were no indications for neurosurgical intervention in the patient.

Each of the newborns after forceps delivery had cranial ultrasound scans (with the exception of four who were discharged from the hospital on the mother's request before examination). In eight cases, subperiosteal haematoma were found, which could be attributed to forceps; however, there were no signs of a scapula bone fracture. In 15 babies, other abnormalities were found that were probably not related to the use of forceps (e.g. single cystis of choroid plexuses, state after the first stage of intraventricular haemorrhage or slightly increased echogenicity requiring further follow-up after discharge) (Table 4).

**Table 4.** Cranial ultrasound scans results of newborns after forceps delivery.

**Cranial ultrasound scans results**	**Number of patents (n)**	**Percent (%)**
Normal	122	83.9
Subperiosteal haematoma	8	5.6
Single cystis of the choroid plexus	5	3.5
State after the 1° IVH	3	2.1
Increased echogenicity (brain parenchyma)	7	4.9

## Discussion

Nowadays, with the continuing increase in caesarean section rates, choosing the right method of delivery is vital not only for maternal and neonatal outcomes after delivery but also for the long-term maternal perspectives during other pregnancies. Most importantly, an instrumental delivery gives the mother higher chances of vaginal delivery in her next pregnancy than a caesarean section [7]. A review of our data showed that in our department, despite a slight increase in the rate of caesarean sections, the frequency of instrumental deliveries over the years has remained relatively stable between 1.8 and 2.8% (average 2.2%). Our results are, therefore, different from the trends reported in other studies, where we observe a continuous decrease in the percentage of instrumental deliveries [1].

As the study showed, the majority of mothers who underwent forceps delivery were primigravidas (n = 116; 78.9 %). Similar results were obtained in the studies by Johnson (75.5%) [8], Pitale (75%) [9] and Okram (73%) [4]. Ages between 26 and 35 concern 72.8 % (n = 107) of the study group. That number correlates with the average age of pregnant women in Poland, according to data of Central Statistical Office. In reviewed comorbidity of patients, it is worth noting that 78.9% (n = 116) of patients had a body mass index (BMI) of 25 and above, with a mean of 27.7. However, the study by Chafika [10] shows no significant BMI difference between women who gave spontaneous birth vaginally or by forceps delivery. Analysing our data and comparing it with the abovementioned, apart from parity, we do not find any significant risk factor for forceps delivery.

In our study group, the most common indications for forceps delivery were foetal distress (82.6%) and maternal exhaustion (12.1%). Although the order of the most frequent indications correlates with the results of study by Johnson [8] (foetal distress 47%, maternal exhaustion 38.5%) and Okram [4] (foetal distress 55%; maternal exhaustion 21%), the percentage distribution requires consideration. Moreover, in another study by Pitale [9], the most common indications for forceps delivery were maternal exhaustion (80%) and foetal distress and prolonged second stage of delivery, both occurring in 10% of the research cases. In our opinion, effective cooperation with the mother in labour and her proper instruction significantly increases the possibility of physiological termination of delivery.

Regarding the maternal outcomes, 77 (58.3 %) women after the forceps delivery experienced injuries or complications (in a few cases, more than one). The most common were vaginal lacerations (38.6%), cervical lacerations (18.2%) and postpartum haemorrhage (13.6%). It is worth noting that all women underwent episiotomies, and after each delivery, the birth canal was thoroughly examined. We did not observe any third- and fourth-degree perineal laceration in the analysed group. Outcomes of our study differs from study by Okram [4] in which the most common complications were: postpartum haemorrhage (5.8%), vaginal and cervical laceration (4.8%) and third- and fourth-degree perineal tear (3.8%). Our results also differ from the study by Johnson [8] where vaginal laceration affected 19%, and third- and fourth-degree perineal tear occurred in 33% and 11.5% of cases studied, respectively. The majority of the newborns (n = 134; 91.2 %) were born in general good condition defined by the Apgar score of more than seven after the first minute of life. Equal or less than 7 points were received by 8.8% of newborns (n = 13). In other publications (e.g., Okram et al. [4], Lebraud et al. [11]), the Apgar score ≤ 7 after the first minute of life was given to between 15% [4] to 19.4% [11] of babies. Regarding the 5^th^ minute Apgar, results of our study were similar to the others (2% of newborns ≤ 7 Apgar's points in our study vs. 2.3% at Okram's [4] and 1.5% at Lebraud's publication [11]). About 7.5 % of newborns required admission to the NICU, which is comparable to other studies (6.7% at Stock's [12] and 7.6% at Bassel's [13]). One newborn was transferred to the therapeutic hypothermy reference center due to the result of umbilical blood gas analysis and neurological symptoms according to the Thompson's scale. However, after an EEG and neurological reassessment, the patient was not qualified for the cooling. After the seventh day of life, the MRI examination was normal, and there were no signs of hypoxicischemic brain damage.

Despite concerns about birth traumas caused by forceps, more than 75% (n = 111) infants included in the study had no signs of injuries. Twenty-three newborns (15.6%) experienced skin damage on the head and face. More severe birth traumas such as clavicle fracture, subperiosteal haematoma or conjunctival haematoma were observed in 13 cases (8.8%). Only one of them needed surgical and neurosurgical consultation due to an extensive subperiosteal haematoma and symptoms of facial nerve palsy. The patient was transferred to the Polish Mother Memorial Hospital. After CT scans and MRI, intracranial and subgaleal haemorrhage and occipital bone fracture were diagnosed. Due to the patient's good general condition and lack of other neurological symptoms, there was no indication for neurosurgery, so non-invasive treatment was provided. The baby was discharged from the hospital after 22 days with a recommendation for further follow-up. Comparing these results with other similar studies can be seen that the total number of injuries in our study was significantly higher [11,12,13]. However, authors of those publications did not consider superficial skin lesions as birth traumas. Only severe injuries such as cephalohaematomas, fractured clavicles or skull fractures were mentioned and concerned from 3.1% to 8.8% of newborns [11,12,13], and these results are comparable to our analysed group.

## Conclusions

Despite the increase in the rate of caesarean sections in our department, the percentage of forceps deliveries remains relatively stable.

Appropriate response to emerging risks in the second stage of labour increases the chances of delivering a newborn in good condition and helps to avoid severe trauma like hypoxicischemic encephalopathy.

Most newborns and mothers after forceps delivery are in general good condition and have no severe traumas.

Forceps delivery should be the skill possessed by every obstetrician and used in daily practice when necessary.
